# Mind the gap (again!): On the challenges for women in physiology

**DOI:** 10.1113/EP092028

**Published:** 2025-02-07

**Authors:** Christina Yfanti

**Affiliations:** ^1^ Centre for Physical Activity Research Rigshospitalet, University of Copenhagen Copenhagen Denmark

This is an article motivated by a conversation with the Editor‐in‐Chief and having read his publication in *Physiology News* entitled, ‘Mind and mend the gap: Women in physiology’ (Bailey, 2023). The account below reflects a very personal journey, not necessarily representing all the employees of the institutions where I worked over the years. Nevertheless, perhaps reaching out and connecting with other women Physiologists with shared experiences will help to highlight the challenges that we face and provide some much needed solutions to the gender and age ‘leaky pipeline’ as described by Professor Bailey. Highlighting the ‘fault lines’ is a good first step towards keeping our important scientific field alive, given that women have more than a minor role to play!

As pointed out in the Editor‐in‐Chief's publication (Bailey, 2023), women are under‐represented in Physiology across a variety of sectors within the field, starting with females not being recruited as participants in human trials (Daitch et al., 2022; Yu, 2021) to female researchers who are often less credited with authorship and females holding fewer tenure track positions and fewer memberships of editorial boards and scientific committees compared with their male counterparts (James et al., 2023; Ross et al., 2022). It is worth noticing that, although the number of women researchers in exercise science and physiology has doubled during the last decade, women still account for <25% of authors in this field (Cowan et al., 2023). This aligns with broader trends across research disciplines, where women comprise only 33% of researchers at the European level (European Commission, 2021). Equally striking is the fact that the proportion of female researchers is higher in younger age groups but declines significantly with age, resulting in a male‐dominated presence in senior academic and leadership positions, particularly among individuals aged ≥55 years (European Commission, 2021). Therefore, this under‐representation of women reveals, apart from a gender gap, also an age gap.

Through my personal journey unfolding in the description below, I am presenting my experience as a mid‐career female Exercise Physiologist who is trying to climb the academic ladder after a career break from academia.

My professional career path until now has been everything but traditional. Transitioning between academia and industry among five different countries, I have been exposed to a diverse range of experiences, acquiring an array of valuable skills that have enriched me both professionally and personally. However, the challenges I have faced have also been numerous. And I am currently facing a new one, for which I have not yet found the solution.

Starting as an undergraduate student in Exercise Physiology, I discovered early on my passion for research in this field. I was lucky enough to have had inspiring and motivational mentors during that time, who introduced me to the world of scientific research and guided me through my initial involvement in the Exercise Physiology field. At that point, I knew I wanted to follow an academic career, continue conducting research and perhaps one day myself become a mentor to guide and inspire young students.

After being successful in my first ever funding challenge, I acquired a PhD scholarship from the Greek State Scholarships Foundation and moved to Denmark to conduct my doctoral studies. My research was focused on the combined effect of chronic aerobic exercise and antioxidant supplements on performance, metabolism and inflammation in healthy humans. My academic journey continued for another couple of years, working as a Postdoctoral Fellow in the USA in the area of brown adipose tissue activation and its implication in human metabolism. Immediately after completing my Postdoc, I reached the first turning point in my personal and professional life. This is when I decided to switch career paths and move to the pharmaceutical industry. What led me to this decision? I suppose the same reasons that lead all academics to make this transition, with the two main ones being income stability and work–life balance.

While I was trying to overcome the challenges within the pharma industry and build a new career from scratch, the thought of how and whether I could go back to academia was very often in my mind. My passion for research, especially in the Exercise Physiology field, had endured. Moreover, my experience in drug development, combining the knowledge with regard to chronic disease pathophysiology and the targeted approach on how to tackle the disease, made me constantly contemplate how Physical Exercise could play a similar role in this. As an Exercise Physiologist, I knew that exercise works as preventive means but also as an adjunct treatment to a vast array of chronic diseases. Apart from that, I was also missing the generation and testing of new ideas, applying hands‐on experimental procedures and having time to think and reflect on processes and results. This is when I reached a turning point for the second time in my professional life and took the decision to move back to academia. However, as both my chronological and academic age were increasing, my chances for returning were becoming slimmer. This is mainly because I was surprised to see that the first eligibility criterion for funding applications was either chronological age or the period since PhD completion, which is usually referred to as someone's ‘PhD age’. Therefore, in this Editorial, the focus is on how PhD age, in combination with a career break, might be a major contributor to the ‘leaky pipeline’ identified before (Bailey, 2023; United Nations Educational SaCo, 2015).

Although I was excluded from many funding opportunities owing to the age limit (either chronological or PhD age), I was excited to discover that I was eligible for the Marie Skłodowska‐Curie Individual Fellowships—Career Restart panel, aimed at individuals who had left academia and aimed to return. Therefore, I took the opportunity, applied, and was very fortunate and immensely thrilled to be awarded a Fellowship post to pursue a randomized controlled trial investigating the effects of exercise training as an adjunct treatment in cancer disease at the Centre for Physical Activity Research in Denmark. Thus, here I am now, more than halfway through my 3‐year fellowship in the area of Clinical Exercise Physiology in Oncology and looking into acquiring funding for my next step. And I would like this next step to be in academia. I want to continue the line of work I am currently persuing, with the ultimate goal of obtaining a faculty position. This is the new challenge that I am currently facing, because my PhD age has become even larger since my initial attempt to return and it continues to be the first eligibility criterion of the funding agencies, at least for funding directed to early career researchers. Considering my number of active years in academia owing to my career break, I qualify as an early career researcher. However, based on my PhD age I am placed in the ‘senior researcher’ category, of course not being able to compete with the publication track record of someone with the same PhD age who never left university. As a result, I am not eligible to apply either for an early career researcher or for a senior researcher funding opportunity!

Coming back to academia after 10 years is an enormous venture, because one needs to start again from the bottom of the academic career ladder. One reason is the fact that the way research in the Physiology field is being conducted has changed over the years. Pre‐registration, use of new methodologies and analyses (e.g., omics) and new monitoring processes are some of these changes that one needs to catch up with while at the same time conducting the main project. The main reason, however, is that, during the 2–3 years that is usually the duration of an entry Fellowship, one is challenged to rebuild an academic curriculum vitae; publish in high‐ranked journals, supervise Master and PhD students, present at conferences, perform dissemination activities, teach, build a network of new collaborators and, very importantly, design new projects and apply (if eligible) to secure funding for the next career step before the current fellowship is completed. However, this seems rather an impossible task to accomplish within the short time frame of 2–3 years, especially when it has to be combined with family and social life. Although this is a challenge that concerns both genders, we can all agree that gender stereotypes that want women to have the primary role in family care still exist, at least across most cultures. There has certainly been a huge improvement to that end since the implementation of extension regulations for parental leave in the eligibility criteria of the main funding agencies, which has contributed significantly towards narrowing the gender gap. Nevertheless, the time spent on maternity leave is usually time with no significant academic productivity (e.g., no publications or data collection). And in most cases, after return from maternity leave, an additional period with low productivity follows because one needs to catch up where one left off and get up to speed before results and outputs are generated and further career progression is achieved. This is time not added to the PhD age, but it plays a great role in the time line for career advancement. Thus, although the PhD age is corrected for maternity leave, in reality it is not corrected in full. Nevertheless, apart from parental leave, we need to take into consideration that a break from academia might have been taken for other reasons, such as other career choices.

And I am, therefore, wondering, why is PhD age so important? What is the rationale behind this being a criterion for someone to continue being a researcher? Does it mean that the younger you are, the more suitable you are to become a scientist? I tend to believe that with age comes maturity, additional skills and wisdom, and one tends to know better how one wants to progress in life. If combined with the experience in diverse working environments, the possibility that this person succeeds in accomplishing their academic and research goals is enhanced. Thus, when such an individual decides to continue into a specific academic career path (or any other path), it is certain that the decision is a conscious, well‐calculated one, with a high probability of success, irrespective of the time since PhD completion. For me personally, experience in drug development and management of clinical trials in the oncology field have broadened my way of thinking with regard to processes, methodologies and outcomes in human studies, which can be applied in the Clinical Exercise Physiology trials.

The PhD age as an eligibility criterion is in place to protect younger researchers competing against established peers in the field who certainly have a larger and, perhaps, more rounded academic track record. It is there to provide the younger scientists with the opportunity to move towards academic independence. And rightfully so. However, PhD age is not necessarily intertwined with academic seniority. It has probably been the case in previous generations, who followed the traditional academic path; however, times have changed. We live in an era where synergy between industry (and other sectors, e.g., clinical experience or experience in governmental bodies) and academia is encouraged, as also reflected in many calls from major funding agencies (Innovation Fund Denmark, 2025; Research & Innovation, 2024; UK Research & Innovation, 2024). Transition of researchers between the two sectors has switched from unidirectional (academia to industry) to bidirectional and is expected to increase more in the future, simply because academia and industry are complementary rather than separate (Bodine, 2011). Substantial funding for research at universities derives from industry, and a significant part of scientific output generated at research institutes is used by industry. Moreover, scientists of the current generation often seek new challenges and opportunities from various sectors to broaden their skillset and experiences. Thus, they are more likely to interchange between university and business/industry positions, given that such a combination will help them to advance both their hard and soft skills (Jacobs, 2024).

Looking through the eyes of a mature female Physiology researcher, with a focus on Clinical Exercise Physiology, I envision to be part of a diverse, inclusive research community of Clinical Exercise Physiologists who will continue providing evidence for the integral role of exercise in the management of chronic disease. Clinical Exercise Physiology has already emerged as a scientific field for some years now. Nevertheless, a vast amount remains to be explored before we are able to provide individualized, targeted exercise interventions for the various patient groups. Enhanced intersectoral (industry, university and government) collaboration is vital for the optimization and further implementation of these interventions into healthcare systems. This synergy is translated both in collaboration and transition of personnel and technical knowhow between sectors and in financial aid that will facilitate these transactions and allow further development. In such effort, PhD age seems to be a hindrance rather than an aid. Usually, the jump from academia to other sectors is relatively easy. The demand and the finances are there.

The question is, how can we facilitate an equally successful inflow of personnel from other sectors back to academia? There are funding agencies that require the addition of an industrial partner in the application process (ABPI, 2025; Innovation Fund Denmark, 2025; Research & Innovation, 2024), often leading to the future transition of the successful grant holder to the industry. Why should not the opposite be possible, for example, when someone completes an industrial PhD to be given the opportunity to obtain a university position? Very importantly, I would argue strongly that funding opportunities tailored specifically to researchers transitioning from early to senior stage or between different sectors are paramount. The majority in this category are women who have taken a break for child or other family care responsibilities. Such funding opportunities will provide these individuals with the ‘extra time’ required for their achievements (publications, supervision, presentations, etc.) from their early career stage to become visible to the broader academic community. Given that a large proportion of researchers in this category are women of a greater chronological age willing to continue in their academic path, the above solutions will allow them to compete on equal terms with their peers, bridge the age gap within the female population in the Physiology field and, ultimately, address the problems with the ‘leaky pipeline’ and the gender bias in Physiology researchers.

## AUTHOR CONTRIBUTIONS

Christina Yfanti and Damian M.Bailley conceived the idea. C. Yfanti wrote the first draft and DM. Bailyprovided feedback. C. Yfanti is accountable for thecontent of the article.

## CONFLICT OF INTEREST

None declared.

## FUNDING INFORMATION

The Centre for Physical Activity Research (CFAS) is supported by TrygFonden (grantsID 101390, ID 20045, ID 125132, and ID 177225) Dr. Yfanti has received funding from the European Union.s Horizon 2020 research and innovation programme under the Marie Sklodowska‐Curie grant agreement No 101029875.

## Data Availability

Data sharing not applicable to this article as no datasets were generated or analysed during the current study.
